# Retraction: SHBG Is an Important Factor in Stemness Induction of Cells by DHT *In Vitro* and Associated with Poor Clinical Features of Prostate Carcinomas

**DOI:** 10.1371/journal.pone.0237487

**Published:** 2020-08-05

**Authors:** The PLOS ONE Editors

Following the publication of this article [1], concerns have been raised regarding the immunoblotting data presented in Fig 3A as well as the immunocytochemistry data presented in Fig 5A.

Specifically,

- In Fig 3A, the bands in Oct3/4 panel 10 nM DHT treated LNCaP cells (lanes 9–12) appear similar to the bands in Nanog panel 0 nM DHT treated LNCaP cells (lanes 1–4).
- In Fig 3A, the bands in GADPH panel 1 nM DHT treated LNCaP cells (lanes 5–8) appear similar to the bands in GADPH panel 1nM DHT treated PC-3 cells (lanes 5–8).
- In Fig 5A, the panel representing immunocytochemical staining of 0 nM DHT treated LNCaP cells appears similar to the panel representing immunocytochemical staining of 0 nM DHT treated PC-3 cells.

The Commission on Research Integrity at Oslo University Hospital and Institute of Clinical Medicine, University of Oslo completed an investigation and confirmed the concerns with Fig 3A and Fig 5A.

The authors indicated that the wrong images have been uploaded for the Nanog panel of the 0 nM DHT treated LNCaP cells, and the GAPDH panel of the 1 nM DHT treated PC-3 cells presented in Fig 3A. Furthermore, the authors indicated that the wrong images have been used for the 0 nM DHT treated LNCaP and PC-3 cells presented in Fig 5A. The authors offered replacement images for the panels of concern and provided supporting data for some of the results reported in the article. However, data were not provided in support of all panels for the experiments in question, and discrepancies were noted between the supporting image provided and the published figure for the Nanog panel of the 0 nM DHT treated PC-3 cells in Fig 3A.

Overall, the data and information provided by the authors have not fully resolved the concerns. Given the remaining issues for Fig 3A and the data shown in the figure, the article’s claims based on the western blot results in this figure are not adequately supported.

In light of the above concerns and in line with the institution’s recommendation, the *PLOS ONE* Editors retract this article.

GW, KA, UA, and ZZ agreed with the retraction. DL and ZS did not confirm agreement or disagreement with the retraction decision. YM, JGW, ES, TS, LV, EP, YY, GK, and JMN either did not respond directly or could not be reached. JL did not agree with the retraction.
